# Exploring Signals on L5/E5a/B2a for Dual-Frequency GNSS Precise Point Positioning

**DOI:** 10.3390/s21062046

**Published:** 2021-03-14

**Authors:** Nacer Naciri, André Hauschild, Sunil Bisnath

**Affiliations:** 1 Department of Earth and Space Science and Engineering, York University, Toronto, ON M3J 1P3, Canada; sbisnath@yorku.ca; 2 German Aerospace Center (DLR), German Space Operations Center (GSOC), 82234 Wessling, Germany; andre.hauschild@dlr.de

**Keywords:** precise point positioning, GPS block IIF, GPS L5 variations, BeiDou-3, Galileo

## Abstract

Due to its nature, Precise Point Positioning (PPP) depends on the GNSS measurements and quality of satellite correction products used to relatively quickly provide precise and accurate positions. With the rapid evolution of Global Navigation Satellite Systems (GNSSs), new frequencies and signals are being broadcast, which have a positive impact on PPP performance. This paper presents, for the first time, a comprehensive analysis of PPP performance from these new GPS, Galileo and BeiDou-2/3 signals, which are not yet commonly used for PPP, with correct mitigation of errors such as the estimation of GPS Block-IIF L5 variations. Satellite orbits and clocks, as well as GPS Block-IIF L5 corrections, are estimated in real-time using DLR’s RETICLE engine, while the user processing is performed with York University’s PPP engine. First, as a reference, PPP performance is assessed on widely used signals: GPS L1/L2, Galileo E1/E5a, and BeiDou-2/3 B1-2/B3. Horizontal and vertical rms of 2.3 and 2.6 cm, respectively, are achieved in static processing and 5.4 and 7.5 cm in kinematic processing after 1 h of processing using real-time satellite correction products. The compatibility of BeiDou-2 and BeiDou-3 on the shared B1-2/B3 frequencies is analyzed and discrepancies in the receiver clock are found. Next, since all three constellations share two common frequencies, the paper focuses on analyzing PPP performance of GPS, Galileo and BeiDou-3 on [L1, E1, B1] at 1575.42 MHz and [L5, E5a, B2a] at 1176.45 MHz. Horizontal and vertical rms of 6.9 and 7.1 cm are achieved in kinematic processing. The effect of the known GPS Block-IIF L5 biases is studied as well, as it is shown to affect the receiver position and clock, as well as the ionospheric estimates and ambiguities. Average improvements of 15% and 20% in the horizontal and vertical rms, respectively, are observed when these biases are mitigated.

## 1. Introduction

The Precise Point Positioning (PPP) augmentation has seen many advances since its early years [1,2]. The technique initially started with forming the ionosphere-free combination of GPS L1 and L2 signals. Results at the time have shown very long convergence times of a few hours [2], which hindered the practicality of the technique. Since then, improvements in the algorithms have led to reduction of the convergence time, as well as betterment of the accuracies. For instance, the use of external atmospheric corrections has been included in the PPP model with a non-negligible impact on the initial errors and convergence time of PPP solutions, as shown in [3,4]. Another area of algorithmic improvement is ambiguity resolution. Indeed, the ability to directly fix the ambiguities to their integer values has led to PPP initial solution convergence only requiring few to tens of minutes as opposed to hours initially [5,6,7].

On top of algorithm advances, PPP has benefited from modernizations of the GNSS constellations [8,9]. GPS originally broadcasted signals on two frequencies L1 and L2 at 1575.42 and 1227.60 MHz, respectively. However, the newer generations of GPS satellites are broadcasting an additional L5 signal at 1176.45 MHz, with higher power and aimed towards safety-of-life applications. Fourteen satellites from the newer generations GPS-IIF and GPS-III have been launched since 2010, all of which broadcast on all three frequencies [10]. Galileo, on the other hand, has been designed to broadcast on four frequencies (E1, E6, E5a, and E5b) with the option to track E5a + E5b together as AltBOC (Alternative Binary Offset Carrier) [11]. The Galileo E1 and E5a frequencies coincide with the GPS L1 and L5 frequencies, respectively. With launches starting in 2011, Galileo is almost complete with satellites E14 and E18 in eccentric orbits set unhealthy but otherwise fully functional. Including these two satellites in the processing leads to a total number of usable satellites of 24 [12]. BeiDou has also been designed to broadcast on multiple frequencies with satellites in Medium Earth Orbit (MEO), Geostationary Earth Orbit (GEO). and Inclined GeoSynchronous Orbit (IGSO). The BeiDou-2 generation is designed to broadcast signals on three frequencies B1-2, B2b. and B3 at 1561.098, 11,207.140, and 1268.52 MHz, respectively. The newer BeiDou-3 generation has been designed to broadcast five frequencies in total (B1, B2a, B2b, B2, and B3), with B1 and B2a coinciding with the GPS L1 and L5 frequencies, respectively. The BeiDou-3 generation has been recently completed, with the last satellites being launched in June 2020 [13]. As of March 2021, the constellation has 44 operational satellites [14]. Given all the modernization of GNSS constellations, the processing of multiple constellations [15,16] and frequencies [17,18] has been shown to greatly improve PPP performance.

With all these new satellites and signals come new challenges. For instance, Wanninger and Beer [19] demonstrated that BeiDou-2 suffers from the existence of satellite-induced, elevation-dependent group delay variations that affect the pseudorange measurements. They provided a satellite-, elevation- and frequency-dependent look-up table to correct for these pseudorange variations.

The GPS Block-IIF L5 signals suffer from clock variations, as first noticed by Montenbruck et al. [20]. These variations are caused by a thermal effect depending on the orbital plane orientation with relation to the Sun. Different strategies are adopted to eliminate these biases: Tegedor and Øvstedal [21] proposed the estimation of separate L5 clocks along with the typical L1/L2 clocks in an offline setting after the first three GPS Block-IIF satellites were launched. Guo and Geng [22] used a similar approach with a focus on triple-frequency processing; Montenbruck et al. [20] proposed a harmonic approximation of the variations; and Li et al. [23,24] estimated the biases based on satellite- and epoch-differenced measurements, where the focus was triple-frequency processing.

BeiDou-2 and BeiDou-3 suffer from the existence of clock and time delay biases between both generations. Jiao et al. [25] showed the existence of these biases between BeiDou-2 and BeiDou-3. They noticed that the biases are receiver-dependent, as some receivers suffer from significant biases, while others do not. Improvements have been noticed when estimating the BeiDou-2 and BeiDou-3 Inter-Signal Biases (ISB) as random constants. Zhang et al. [26] showed that not taking these ISBs into account affects the Signal-In-Space Ranging Errors (SISRE) of the satellite orbits, while Li et al. [27] showed their effect on both receiver and satellite DCB estimation.

This paper analyzes the current performance of GPS, Galileo and BeiDou on these new signals with correct mitigation of errors including the BeiDou-2/3 ISBs and the GPS L5 variations. To do so, GPS, Galileo and BeiDou-2/3 satellite orbit and clock products, as well as GPS Block-IIF L5 variations, are estimated in real-time using DLR’s RETICLE engine. DLR contributes its real-time products to the real-time service of the IGS together with several other analysis centers from North-America, Asia, Europe and Australia. The individual contributions are combined into the correction data stream of the IGS Real-Time Service (RTS) [28], which provides PPP corrections via the internet free of charge. More details can be found on the IGS website (https://www.igs.org/rts/ (accessed on 3 March 2021)). The user processing is performed with York University’s engine, which is a multi-frequency multi-constellation capable software [29,30].

This paper is divided into three main sections. First, a description of the product generation using the RETICLE engine is given. Then, PPP performance is analyzed on widely used signals for GPS (L1/L2), Galileo (E1/E5a) and BeiDou-2/3 (B1-2/B3), followed by an analysis of PPP performance of all three constellations on the same two frequencies [L1, E1, B1: 1575.42 MHz] and [L5, E5a, B2a: 1176.45 MHz]. In addition to these three main sections, additional analysis is performed: analysis of the inter-operability of BeiDou-2 and BeiDou-3 on the user side; analysis of the effect of the GPS Block-IIF L5 biases on the estimated PPP states; and how correcting for these biases affects the PPP position. The paper ends with conclusions and thoughts for future.

## 2. Real-Time Clock Estimation with RETICLE

The Real Time Clock Estimation (RETICLE) engine is real-time software that processes input from data streams with GNSS observations and produces precise estimates of clock offset and drift for each GNSS satellite along with other parameters using a federated Kalman-filter. These estimates are output with a delay of only a few seconds. The system is capable of processing more than 100 stations and all satellites of GPS, GLONASS, Galileo, BeiDou-2/-3, and QZSS. The following sections provide an overview of the design of the RETICLE processing engine.

### 2.1. Overview

The RETICLE engine relies on multi-threaded processing using a federated Kalman-filter design to process real-time data from a global GNSS reference station network. For each station of the network, pseudorange, carrier-phase, and carrier-to-noise-density ratio (C/N_0_) observations are processed in a local station filter. Each filter processes single, uncombined observations and can process any number of different frequencies and signals for each constellation. The local station filters operate at an update rate of 1 Hz.

A global filter merges the satellite clock offset estimates from the local filters into a combined solution. This global merging filter parameterizes the clock offset and drift for each GNSS satellite. The result of the global clock combination filter is then fed back into the local filters. The global clock merging filter operates at a reduced rate of once every 5 s for processing performance reasons.

### 2.2. Station Filter Description

**Figure 1:**
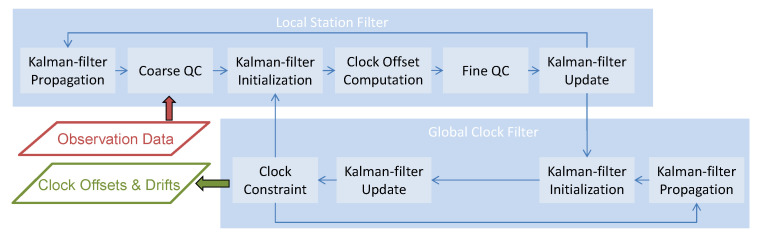
Processing steps of the local station filters and the global clock merging filter in RETICLE.

In this section, the data processing in the individual station filters is described in more detail. A flowchart with the different processing steps in a local station filter and its interaction with the global merging filter is shown in Figure 1. When a new epoch with observation data for a reference station is available from the real-time data stream, the observation data, together with orbit predictions and all necessary meta-data, is passed to the corresponding local station filter for processing. If the Kalman-filter has already been initialized and has processed data, the filter state is predicted to the epoch of the new observation data.

The first processing step is a coarse quality control (QC) of the new observation data to reject measurement outliers and remove observations that either cannot be processed due to incomplete meta-data or have been excluded from the processing in the configuration.

The next step is the initialization of the Kalman-filter state vector in case new estimation parameters have become observable through the measurements. Since the visibility of the GNSS satellites changes over time for each reference station, new estimation parameters, for example satellite clock offsets or slant ionospheric delays, or ambiguities must be added to the state vector from time to time. Similarly, when GNSS satellites vanish from the antenna’s field-of-view, the corresponding state vector parameters are removed from the filter state.

After the initialization of the filter state, a pseudorange-based single positioning solution of the station coordinates and clock offset is computed using dual-frequency observations that have passed the data screening. Bad pseudorange measurements that fail a χ-square residual test are recursively rejected. The computed clock offset is used as a a priori value during the following quality control step and the Kalman-filter update.

The next step is a second data quality control, during which all valid observations are used, together with the local filter’s estimates, to compute measurement residuals and perform another χ-square test. Contrary to the first test, un-combined pseudorange and carrier-phase measurements are used this time. Observations which fail the χ-square test are recursively rejected.

After the second data quality control, the Kalman-filter is updated using all pseudorange and carrier-phase measurements which have not been rejected. Single, un-combined observations are used for the update. After the successful filter update, the state vector and covariance information are passed to the global clock filter, for merging the satellite clocks with the results from other local filters. More details on the merging process are provided in the following section. This last step concludes one complete station filter update cycle. As new measurements arrive for this station, the Kalman-filter state is predicted to this epoch and the processing loop starts again.

The local filter state vector comprises one satellite clock offset per GNSS satellite and one receiver clock offset per constellation. The clock offsets refer to an ionosphere-free combination of two signals, which are referred to as clock reference signals in the following.

Atmospheric delays are accounted for by estimating ionospheric and tropospheric delay parameters. For the ionosphere, one slant delay (or STEC parameter) per satellite is estimated. The filter state also comprises one zenith tropospheric delay (ZTD) parameter. Even though a model of the standard troposphere is used to correct the observations for the corresponding slant delay, this additional ZTD correction parameter is necessary to compensate for differences of the actual delay to the empirical model.

Bias and ambiguity parameters in the observable must also be estimated. For each pseudorange observation, which differs from the selected clock reference, a differential code bias (DCB) is estimated. For simplicity, the receiver- and the satellite-dependent contribution are lumped into one combined estimation parameter per satellite. For each un-combined carrier-phase observation a float ambiguity parameter is estimated, which contains the integer ambiguity, as well as the satellite- and receiver-dependent fractional carrier-phase biases (FCBs).

Special modeling is necessary for the L5 signals of GPS Block-IIF satellites. It was found by Montenbruck et al. [20] that systematic variations are present between a clock solution referenced to the L1 and L2 signals and a L1/L5 clock due to thermal line bias variations. The magnitude of the variations depends on the relative orientation of the satellite’s orbital plane to the Sun direction and has a maximum peak-to-peak variation of approximately 1 ns. To compensate for this effect when L5 observations from these satellites are processed, a second clock offset correction parameter is estimated. This additional clock term can be used to make the estimated L1/L2 satellite clock either consistent with an ionosphere-free L1/L5 clock or allow proper modeling for un-combined triple-frequency processing. For GPS Block-IIF satellites, the local station filters and the global merging filter actually contain two clock states per satellite in case L5 observations are processed.

### 2.3. Clock Fusion Filter Description

The global clock fusion filter processes the estimated clock offsets and covariance information from the local station filters and combines this information into a global, merged estimate. All local filters pass their state and covariance information to the merging filter after the Kalman-filter update as shown in Figure 1. The estimated clock offsets are treated as “pseudo”-observations by the merging filter. The global merging filter initializes missing clock states in case prior observations were not available. If the clock filter has been initialized and processed measurements, the Kalman-filter state vector has been predicted to the current epoch in the previous step. After prediction or initialization, the estimates of the clock offsets are processed in the Kalman-filter update into a merged global solution. In the last step, a GNSS clock constraint is applied, which is necessary to align the average GNSS clock offsets to a reference and prevent a common global shift in all GNSS satellite clocks and the corresponding receiver clocks. This clock alignment is realized by constraining the average estimated clocks to the average clock of the broadcast ephemerides for each GNSS individually. Thus, the mean GNSS satellite clocks are aligned to the respective GNSS system time. If necessary, group delay corrections from broadcast ephemeris data, as well as DCBs, are used to account for differences in the selected reference signals between RETICLE and broadcast clocks. The GPS timing group delay (TGD) is the bias between the L1 and L2 P(Y) code signals that is transmitted in the broadcast navigation message. The Galileo broadcast group delay (BGD) is the equivalent of the TGD for the E1/E5a and E1/E5b signals, respectively.

The GNSS satellite clocks in the merging filter are modeled using the clock offset and the clock drift (or frequency offset). This first-order model increases the accuracy of short-term clock predictions, which is especially useful when extrapolating the correction values into the future, for example, in real-time PPP applications.

### 2.4. Modeling of GNSS Observations

The single-frequency observations for pseudorange $p_{u,j}^{s}$ and carrier-phase $\varphi_{u,j}^{s}$ for satellite *s*, receiver *u*, and signal *j* are modeled according to

(1) $$\begin{array}{cc} p_{u,j}^{s}= & \rho_{u}^{s}+c\left(d{\hat{t}}_{u}-d{\hat{t}}^{s}\right)+c\left({\hat{d}}_{j}^{s}+{\hat{d}}_{u,j}\right)\\ \; & +k_{j}{\hat{I}}_{u,1}^{s}+m_{u}^{s}T_{u}+e_{u,j}^{s} \end{array}$$

(2) $$\begin{array}{cc} \varphi_{u,j}^{s}= & \rho_{u}^{s}+c\left(d{\hat{t}}_{u}-d{\hat{t}}^{s}\right)+\lambda_{j}A_{u,j}^{s}\\ \; & -k_{j}{\hat{I}}_{u,1}^{s}+m_{u}^{s}T_{u}+\epsilon_{u,j}^{s} \end{array}$$

with the modeled range $\rho_{u}^{s}$ between GNSS satellite position $r^{s}$ and user position $r_{u}$. The other parameters are the receiver clock offset $d{\hat{t}}_{u}$, the satellite clock offset $d{\hat{t}}^{s}$, speed of light *c*, the mapping function for wet tropospheric delay $m_{u}^{s}$, the wet tropospheric zenith delay $T_{u}$, the slant ionospheric delay ${\hat{I}}_{u,1}^{s}$ on the frequency of the reference signal 1, the ionospheric frequency factor $k_{j}=f_{1}^{2}/f_{j}^{2}$ to convert the ionospheric delay on the frequency $f_{j}$ to the delay on the reference signal’s frequency $f_{1}$, the code biases for satellite ${\hat{d}}_{j}^{s}$ and receiver ${\hat{d}}_{u,j}$, the wavelength $\lambda_{j}$, and the float carrier-phase ambiguity $A_{u,j}^{s}$, which also contains the phase biases, and the receiver noise and multipath for pseudorange $e_{u,j}^{s}$ and carrier phase $\varepsilon_{u,j}^{s}$. It should be noted that some parameters are omitted from (1) and (2) to achieve briefness of notation: the relativistic correction, the combined effect of transmitter and receiver phase-center offset and variations, the dry tropospheric delay, the carrier-phase wind-up effect, and group delay variations are assumed to be eliminated through modeling.

In the above notation, the satellite and receiver clock offsets refer to a dual-frequency ionosphere-free combination of two reference pseudorange observations on different frequencies, which are referred to as signal 1 and 2. As a result, both $d{\hat{t}}_{u}$ and $d{\hat{t}}^{s}$ consist of the true receiver and satellite clock offsets $dt_{u}$ and $dt^{s}$ biased by the receiver and satellite code biases $d_{u}$ and $d^{s}$ on these reference pseudoranges:

(3) $$\begin{array}{cc} d{\hat{t}}^{s} & =dt^{s}-\left(\alpha d_{1}^{s}+\beta d_{2}^{s}\right) \end{array}$$

(4) $$\begin{array}{cc} d{\hat{t}}_{u} & =dt_{u}+\left(\alpha d_{u,1}+\beta d_{u,2}\right) \end{array}$$

where α and β are the coefficients of the ionosphere-free dual-frequency combination. Furthermore, the true ionospheric slant delay $I_{u,1}^{s}$ is biased by the differential code bias (DCB) for the receiver $d_{u,21}=d_{u,1}-d_{u,2}$ and the satellite of the reference signals:

(5) $${\hat{I}}_{u,1}^{s}=I_{u,1}^{s}+c\beta \left(d_{u,21}+d_{21}^{s}\right)$$

The DCB terms for satellites ${\hat{d}}_{j}^{s}$ and receivers ${\hat{d}}_{u,j}$ in Equation (1) consist of a combination of different DCBs:

(6) $$\begin{array}{cc} {\hat{d}}_{j}^{s}= & \beta \left(1-k_{j}\right)d_{21}^{s}-d_{j1}^{s} \end{array}$$

(7) $$\begin{array}{cc} {\hat{d}}_{u,j}= & \beta \left(1-k_{j}\right)d_{u,21}-d_{u,j1} \end{array}$$

It should be noted that if the clock reference pseudoranges on the signals 1 or 2 are used in Equation (1), the DCB terms in Equations (6) and (7) yield zero in both cases, as DCBs are not required when these pseudoranges are processed. Only if other signals are used will the bias term remain.

In the special case of GPS L5 observations from Block IIF satellites, a pure DCB correction is not sufficient due to the aforementioned thermal line bias variations. In this case, an additional time-variable clock correction must also be applied, which is provided by the RETICLE system as an additional delta-clock correction $\Delta t_{5}^{s}$ at the same rate as the L1/L2 clock offset estimate. This term must be added to the L1/L2-compatible satellite clock correction to achieve a L1/L5-compatible satellite clock correction $d{\hat{t}}_{5}^{s}$ as follows:

(8) $$\begin{array}{c} d{\hat{t}}_{5}^{s}=d{\hat{t}}^{s}+\beta \Delta t_{5}^{s} \end{array}$$

Substituting Equation (8) into Equations (1) and (2) yields the following observation equations for the GPS Block IIF L5 observations:

(9) $$\begin{array}{cc} p_{u,5}^{s}= & \rho_{u}^{s}+c\left(d{\hat{t}}_{u}-d{\hat{t}}_{5}^{s}+\beta \Delta t_{5}^{s}\right)+c\left({\hat{d}}_{5}^{s}+{\hat{d}}_{u,5}\right)\\ \; & +k_{5}{\hat{I}}_{u,1}^{s}+m_{u}^{s}T_{u}+e_{u,5}^{s} \end{array}$$

(10) $$\begin{array}{cc} \varphi_{u,5}^{s}= & \rho_{u}^{s}+c\left(d{\hat{t}}_{u}-d{\hat{t}}_{5}^{s}+\beta \Delta t_{5}^{s}\right)+\lambda_{5}A_{u,5}^{s}\\ \; & -k_{5}{\hat{I}}_{u,1}^{s}+m_{u}^{s}T_{u}+\epsilon_{u,5}^{s} \end{array}$$

## 3. Multi-GNSS, Real-Time Precise Point Positioning

The following section provides the results of the study. A description of the processing strategy on the user side is provided first, followed by results from processing GPS + Galileo + BeiDou-2/3 on commonly used frequencies. Then, the results from processing GPS + Galileo + BeiDou-3 on two shared frequencies are presented.

### 3.1. Algorithm Description

York University’s York-PPP engine is used to process IGS stations using the DLR products described above at the user side. The engine is capable of multi-GNSS, multi-frequency processing. Table 1 highlights the strategy used in the estimation and correction of various PPP parameters.

The reference coordinates for the processed datasets are the IGS SINEX positions. The processing is performed in un-combined mode, meaning that linear combinations of measurements are not formed and that the raw measurements on each frequency are processed. Additional corrections such as the Earth rotation, phase windup, relativistic effects, solid earth tides, etc. are performed following the IERS conventions [34]. The IGS14 ANTEX corrections are used to correct for satellite antenna errors for GPS, Galileo, and BeiDou on all the used frequencies. However, since GPS L5 antenna corrections are not available in the IGS ANTEX file, the L2 corrections are applied for the L5 signals. Similarly, ANTEX corrections are not available for the BeiDou-3 B1 and B2a signals. B1-2 and B2b corrections are applied in that case. Due to the existence of Inter-System Biases (ISB), one receiver clock parameter is used for each constellation. All receiver clocks are estimated as white noise and are independent of one another. As discussed in Section 3.2, BeiDou-2 and BeiDou-3 have been processed as if they were different constellations at the user-side. This distinction means that each BeiDou generation has a different receiver clock. Therefore, in total, four receiver clocks are estimated: GPS, Galileo, BeiDou-2, and BeiDou-3. Although Galileo satellites E14 and E18 are on eccentric orbits, they were deemed functional and included in the processing.

The measurements are weighted differently depending on their elevation angle and a constellation-specific weighting factor. The weighting scheme in Equation (11) is used. $\frac{1}{\sigma_{P}^{2}}$ and $\frac{1}{\sigma_{\Phi }^{2}}$ are the pseudorange and carrier-phase weights, respectively. $\sigma_{P_{90}}$, $\sigma_{\Phi_{90}}$, and *E* are the measurement noise at zenith for pseudorange and carrier-phase measurements and the elevation angle, respectively. $\sigma_{P_{90}}$, $\sigma_{\Phi_{90}}$, and *a* are set to 3 dm, 3 mm, and 0.15, respectively. Due to the difference in the quality of signals between constellations, a weighting factor $\sigma_{C}$ is derived from the accuracy of the real-time products for each GNSS constellation. The weighting factors reflect systematic clock biases caused by receiver-dependent pseudorange biases. Such biases would be absorbed by the carrier-phase ambiguities, which is why the carrier-phase observations are not de-weighted. The weighting values are summarized in Table 2.

(11) $$\left\{\begin{array}{cc} \sigma_{P} & =\frac{\sigma_{P_{90}}}{a+(1-a)sinE}+\sigma_{C}\\ \sigma_{\Phi } & =\frac{\sigma_{\Phi_{90}}}{a+(1-a)sinE} \end{array}\right.$$

Moreover, BeiDou-2’s IGSO and MEO satellites suffer from group delay variations, as seen in [35]. To remedy the effect of these variations, which can reach values up to 1 m, the frequency- and elevation-dependent corrections provided in [19] are applied on both the network and user sides.

**Figure 2:**
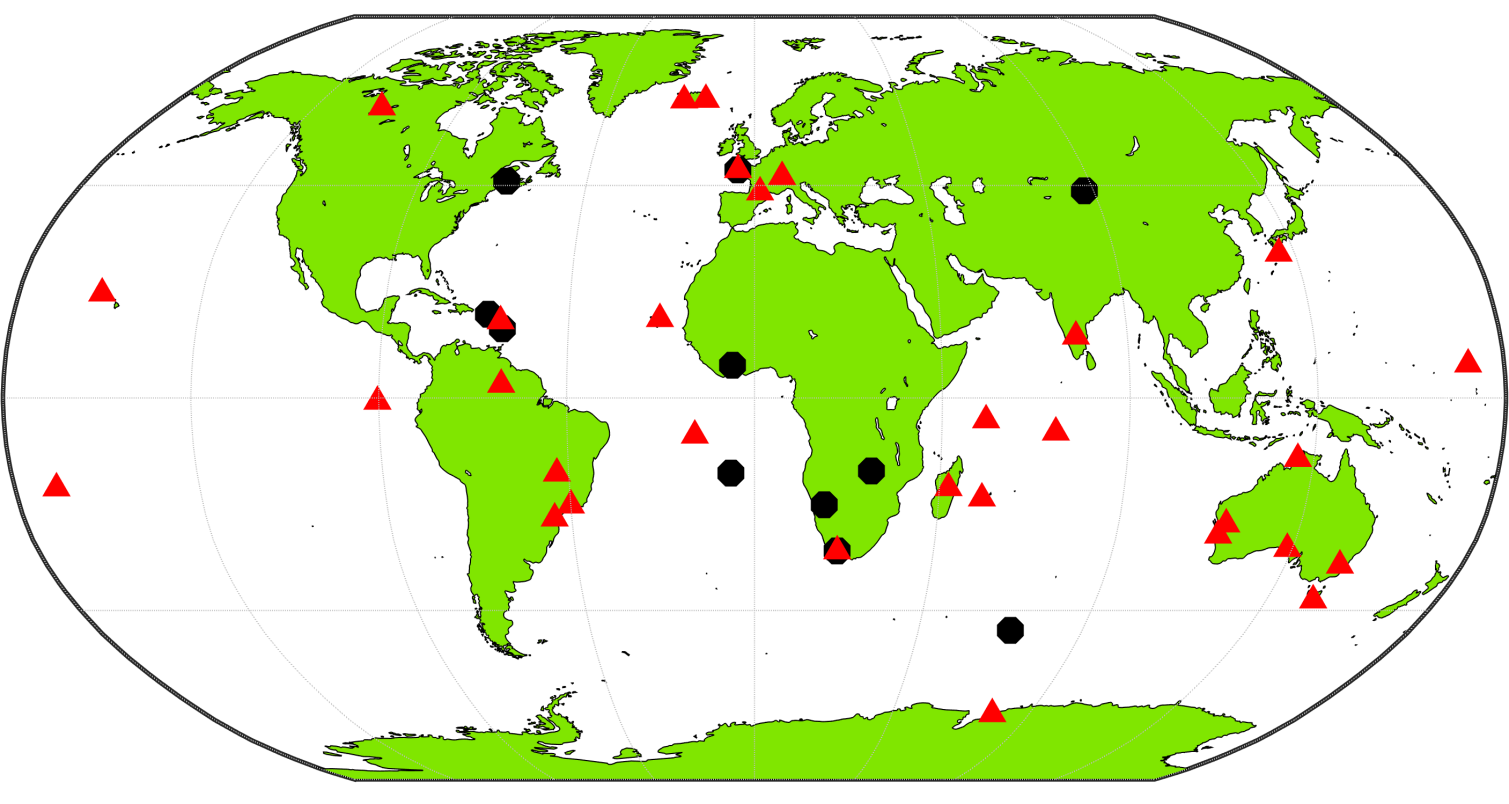
Distribution of stations used in the processing. Red dots are stations used for the multi-GNSS analysis. Black dots are stations used in the GPS L5 analysis.

For the following analysis, two sets of IGS stations are selected and processed on seven days, between DoY 183 and 189, 2020, as shown in Figure 2:

- Thirty-three worldwide IGS stations are used for the multi-GNSS analysis, shown in red in Figure 2. This set of stations is chosen randomly, as long as the station provides day-long observations from GPS, Galileo, and BeiDou. The signals used with these stations are L1/L2 for GPS, E1/E5a for Galileo, and B1-2/B3 for BeiDou-2 and -3.
- Eleven IGS stations are used for the L1/L5 analysis, shown in black in Figure 2. These stations are chosen by ensuring that they provide observations from the novel B1 and B2a BeiDou-3 signals, as well as GPS L5 measurements. Additionally, due to the limited number of stations observing these signals, they are split into 6 h sessions, and only the sessions with more than four visible GPS IIF satellites are kept for processing. The processed signals are L1/L5 for GPS, E1/E5a for Galileo, and B1/B2a for BeiDou-3; therefore, both frequencies are the same for all three constellations: 1575.42 and 1176.45 MHz.

### 3.2. Combined GPS, Galileo and BeiDou Processing on Common Frequencies

The following section assesses the performance of GPS, Galileo, and BeiDou-2/3 on their commonly used frequencies. The compatibility between BeiDou-2 and BeiDou-3 is investigated.

#### 3.2.1. Multi-GNSS Analysis

In this section, the performance of the GPS, Galileo, BeiDou-2, and BeiDou-3 constellations with the L1/L2, E1/E5a, and B1-2/B3 frequencies is assessed. To do so, five constellation combinations are chosen, as highlighted in Table 3. All possible combinations are analyzed except two: Galileo-only and BeiDou-only solutions are not included in the combinations. This exclusion is due to the limited number of satellites that can be seen at the chosen ground stations from these constellations. Indeed, there are numerous epochs where fewer than four satellites from the individual constellation are tracked, rendering PPP unfeasible. Therefore, only combinations of these constellations with at least one other constellation are kept. GLONASS is also not included in the processing, as it does not have an L5-compatible signal and would therefore not be useful in the following sections’ analysis.

**Figure 3:**
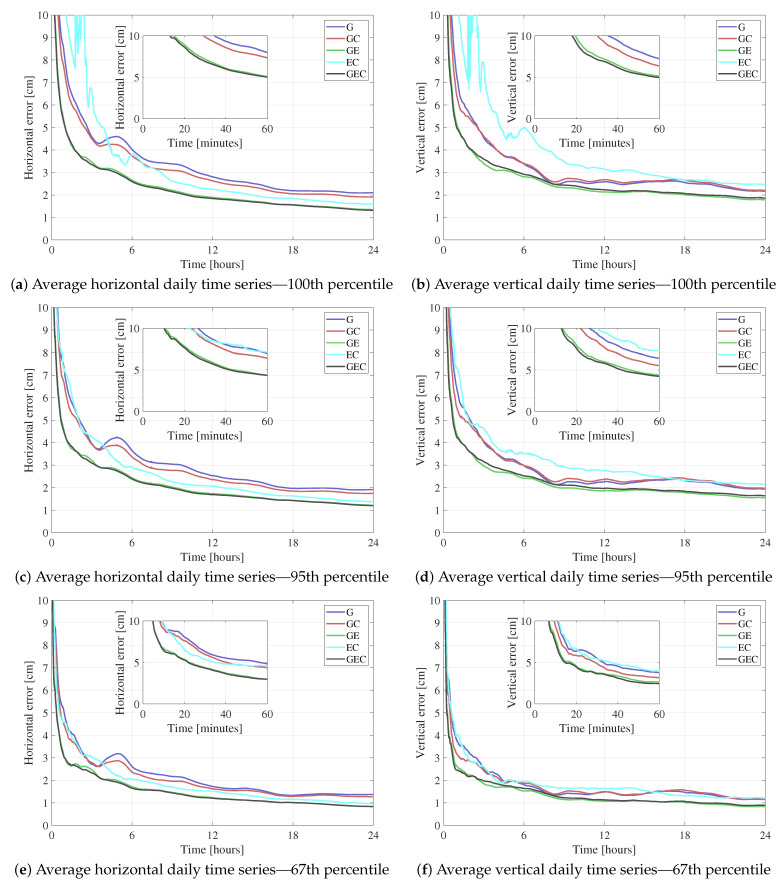
Average horizontal and vertical errors for different constellation combinations at the 100th, 95th, and 67th percentiles.

**Figure 4:**
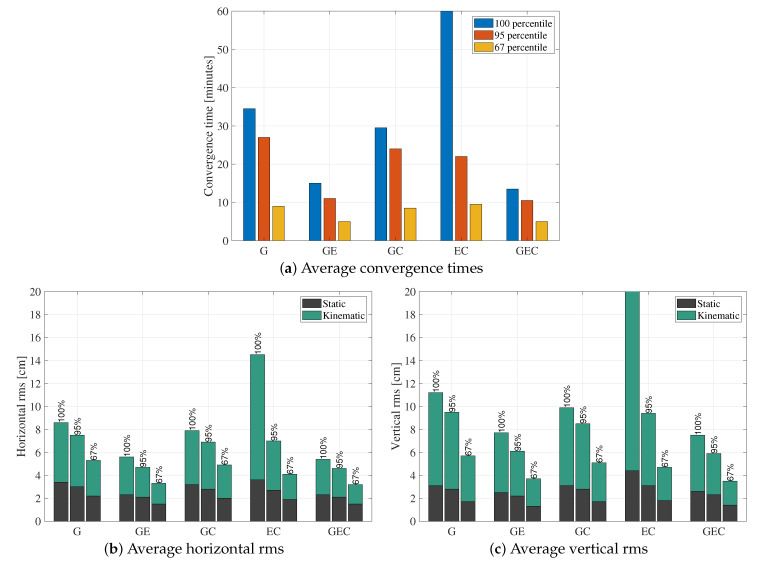
Convergence time (**a**); horizontal rms (**b**); and vertical rms (**c**) corresponding to Figure 3 results—100th percentile EC convergence time and vertical rms exceed the limits of the *y*-axes.

Figure 3 showcases the average results from processing all 33 red stations in Figure 2 for a week, in static mode. The figure shows the epoch-by-epoch average for the horizontal and vertical errors at three different percentiles: 100th, 95th, and 67th. Figure 4 summarizes the statistics of the graphs in Figure 3. The convergence time is defined as the time it takes for the horizontal error to reach and settle below 10 cm. The root mean square (rms) error is computed from the first to the twenty-fourth hour in order to highlight the accuracy after convergence.

**Figure 5:**
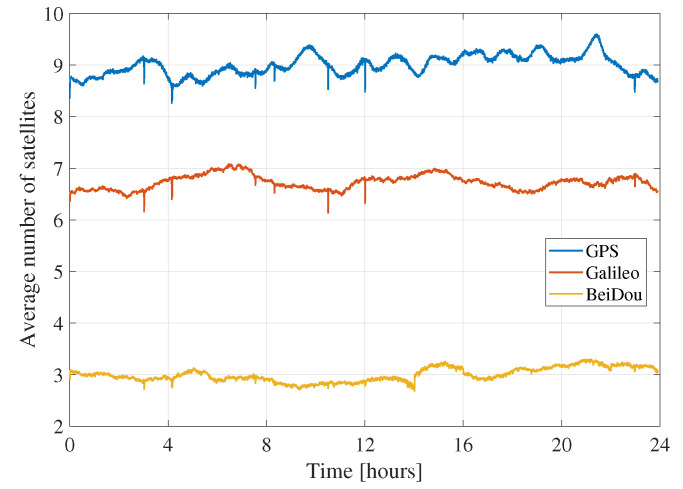
Epoch-by-epoch mean number of GPS, Galileo and BeiDou satellites across all 33 stations over seven days.

**Figure 6:**
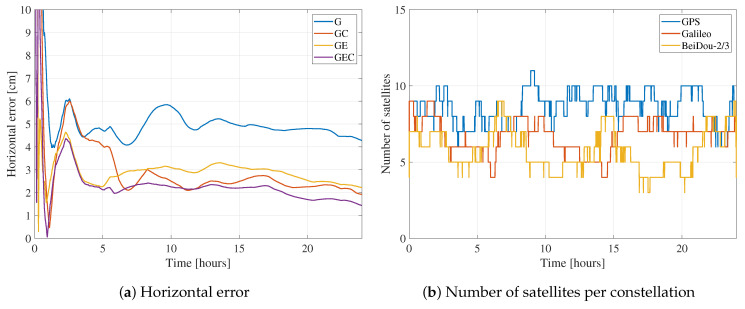
Horizontal error and number of satellites for station SUTH on DoY 187, 2020 for different constellation combinations.

When it comes to the constellation combinations containing GPS, these results indicate that the GPS-only solution has higher rms and convergence time compared to GE, GC, and GEC. This behavior is expected, as adding constellations improves the geometry and increases the number of measurements, which is helpful in a technique that is as measurement-dependent as PPP. On the other hand, GE results have lower horizontal and vertical rms and convergence time compared to GC. This behavior means that, with regards to a GPS-only solution, adding Galileo improves the solution more than adding BeiDou. The most probable reason behind this difference is that BeiDou has a lower number of visible satellites globally compared to Galileo. Figure 5 shows the epoch-by-epoch average of the number of processed satellites from all three constellations, based on all processed datasets. Although this number of satellites time series does not represent the number of satellites at a specific location or on a specific day, it is helpful to show that, on average, only three BeiDou satellites are processed compared to averages of seven and nine for Galileo and GPS, respectively. Note that BeiDou GEO satellites are not included in the processing, and that some stations have a low number of visible BeiDou satellites, as not all stations tracked BeiDou-3 signals. This low number of BeiDou satellites would explain the GC performance compared to GE. On the other hand, GEC solutions are very close to GE in terms of performance. To understand the reason behind this behavior, the results from one of the datasets with a relatively big number of BeiDou satellites are shown in Figure 6. The dataset has six BeiDou satellites on average, compared to nine for GPS and seven for Galileo. Both BeiDou and Galileo improve the GPS-only solution. However, minimal differences exist between both GE and GC solutions and the GEC solution. This limited impact of the third constellation could be due to the fact that the average number of satellites is already high enough with two constellations that an additional one does not have significant effect.

When only processing Galileo and BeiDou, it appears that both horizontal and vertical components at the 100th percentile are noisy and have several jumps, leading to relatively high rms and a convergence time which reaches 146.5 min. This behavior is due to some outlier datasets having a very low number of visible satellites because of the location of the station, time of day, low elevation angle, etc. Such datasets have high errors, which adversely affect the average solutions. These outliers are eliminated in the 95th and 67th percentiles, where the EC combination is comparable to the other combinations.

The datasets have also been processed in kinematic mode with position state process noise typical of a car (120 km/h), in order to assess the performance when the position is not averaged out throughout the processing. Doing so leads to more variations in the position estimates, and therefore slightly higher rms. The rms from the kinematic processing are summarized in Figure 4, along with the static rms. It is clear that kinematic processing leads to slightly higher rms in both horizontal and vertical components. When it comes to comparing the different constellation combinations, the same conclusions hold compared to the static case. One interesting observation is that, in static mode, the horizontal and vertical rms have very close values. In some instances, the vertical rms is smaller than the horizontal one, which goes against the fact that the vertical component cannot be estimated as well as the horizontal one due to the satellite geometry, due to all satellites being above the receivers. Such behavior could be due to the averaging power of static processing. However, in kinematic processing, the vertical components are a few centimetres higher than the horizontal ones, which is more realistic and expected.

#### 3.2.2. BeiDou-2 and BeiDou-3 Compatibility

**Figure 7:**
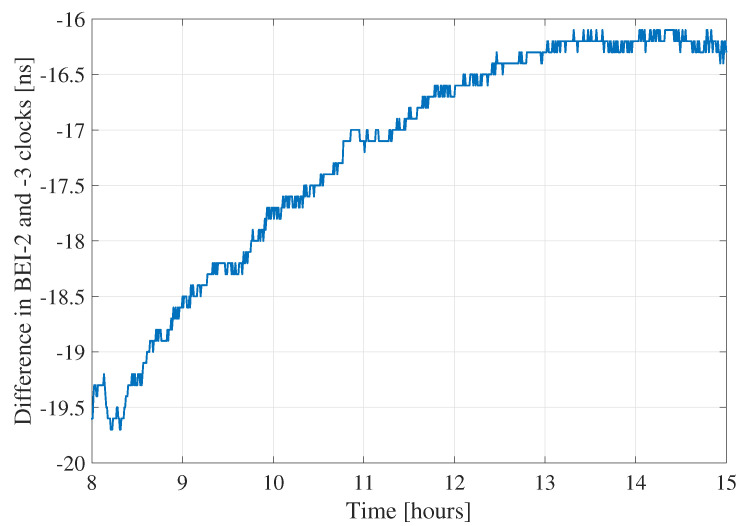
Difference between the estimated BeiDou-2 and -3 receiver clocks for station TLSE on DoY 183 between UTC Hours 8 and 15.

**Figure 8:**
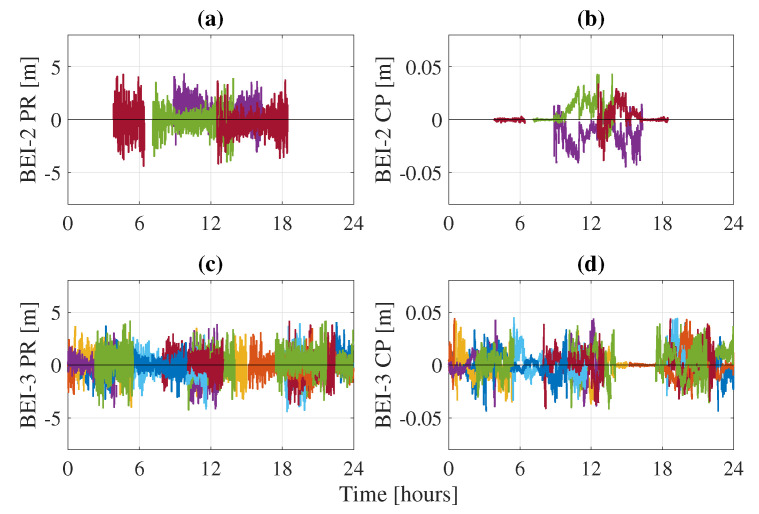
BeiDou-2 pseudorange PR (**a**) and carrier-phase CP (**b**) residuals and BeiDou-3 pseudorange PR (**c**) and carrier-phase CP (**d**) residuals for station TLSE on DoY 183 with separate receiver clocks for each generation.

**Figure 9:**
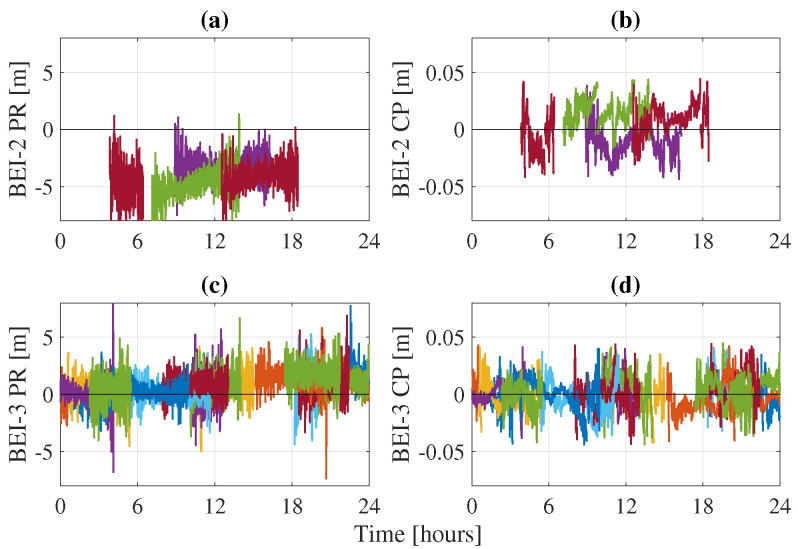
BeiDou-2 pseudorange PR (**a**) and carrier-phase CP (**b**) residuals and BeiDou-3 pseudorange PR (**c**) and carrier-phase CP (**d**) residuals for station TLSE on DoY 183 with a common receiver clock for both generations.

The BeiDou constellation suffers from the presence of biases between both its generations. Indeed, clock and TGD biases have been observed between BeiDou-2 and BeiDou-3. These biases affect the quality of the orbits, as well as the estimation at the user side and are receiver- and frequency-dependent. Zhang et al. [26] computed these values for different types of receivers and on different frequencies. To remedy to the issue, one can either directly apply these values as corrections or process both BeiDou generations as two different constellations by estimating a different receiver clock for each. Applying the corrections at the network side leads to a decrease of BeiDou-3 SISRE. On the user side, a different receiver clock is used for BeiDou-2 and BeiDou-3. Figure 7 shows an example of the difference between the BeiDou-2 and BeiDou-3 receiver clocks when estimated separately for station TLSE. The difference between both clocks contains the biases between the two BeiDou generations, which can reach relatively high values of 20 ns or 6 m. If unaccounted for, these TGD biases go into the residuals, as they cannot be fully absorbed by the other parameters of the PPP model. Figure 8 and Figure 9 show the residuals from GEC processing of station TLSE on DoY 183 with a common receiver clock for all BeiDou satellites and with separate clocks for BeiDou-2 and BeiDou-3. It is clear that using a common clock for both BeiDou generations leads to biases in the residuals: the BeiDou-2 pseudorange residuals are not centered around zero but rather around 4/5 m. On the other hand, the BeiDou-3 pseudorange residuals are centered around zero until Hour 9 where the residuals start shifting and end up being centered around 2 m after Hour 15. Therefore, it is necessary to account for these TGD biases as they affect the PPP model since they not only appear in the residuals but can also affect the estimation of the PPP parameters.

### 3.3. Combined GPS, Galileo, and BeiDou Processing on L1/L5 Frequencies

In this section, the performance of GPS, Galileo and BeiDou-3 on the same two frequencies is assessed. The two frequencies are [L1, E1, B1] at 1575.42 MHz and [L5, E5a, B2a] at 1176.45 MHz. Due to the limited number of GPS satellites broadcasting the L5 signal, a different set of stations from the one in Section 3.2 is used in this section. Indeed, datasets from the dark-colored stations in Figure 2 are split into 6 h sessions. These stations are chosen by making sure that they specifically log measurements from the desired frequencies for all three constellations. The 6 h sessions are further filtered and only those with more than four visible GPS IIF satellites are selected and used in the processing for this section. This condition is set to have enough GPS satellites to influence the solutions and, more specifically, to assess the effect of the GPS L5 bias on the estimation of the parameters. However, the minimum number of GPS satellites condition does not mean that all four GPS satellites can be used in the processing as some would have low elevation angles, while others could be rejected by quality control measures.

#### 3.3.1. Effect of GPS L5 Biases

**Figure 10:**
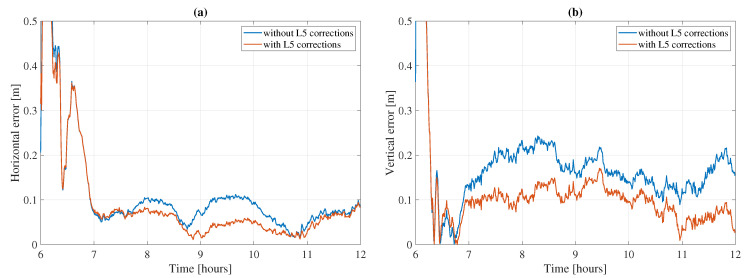
Average horizontal (**a**) and vertical (**b**) errors for station CRO1 on DOY 186, 2020 between UTC Hours 6 and 12. GEC constellations processed in kinematic mode on [L1,E1,B1]/[L5,E5a,B2a] frequencies, with and without the application of the GPS L5 corrections.

First, the effect of the known GPS L5 biases on the positioning solution is assessed on a sample dataset. To do so, GPS measurements on the L1/L5 frequencies are processed using two sets of satellite clocks: L1/L2 clocks on which L5 corrections are not applied, and L1/L5 clocks, which are the same L1/L2 clocks to which the L5 corrections are applied. Due to the number of GPS satellites occasionally dropping below four satellites and to stabilize the positioning solution, Galileo and BeiDou-3 are processed as well. Figure 10 and Table 4 show the effect of accounting or not for the GPS L5 biases on station CRO1, which was processed with GPS, Galileo, and BeiDou-3 on DOY 186, 2020 between Hours 6 and 12 UTC in kinematic mode. Galileo and BeiDou-3 are processed on the same frequencies and with the same setting in both cases.

Looking at station CRO1’s results, it is clear that accounting for the GPS L5 biases and correcting them improves the positioning solution. Both horizontal and vertical components are improved as, demonstrated by the time series as well as the rms. Indeed, when correcting the L5 biases, the horizontal rms decreases from 18.9 to 11.6 cm while the vertical rms decreases from 17.3 to 10.2 cm. The convergence time, being defined as the time it takes for the horizontal error to settle below 10 cm, is much bigger (235.5 min) when using L1/L2 clocks as opposed to 56.5 min when using L1/L5 clocks. This big convergence time is due to the horizontal solution going slightly above the 10 cm threshold between Hours 9 and 10.

**Figure 11:**
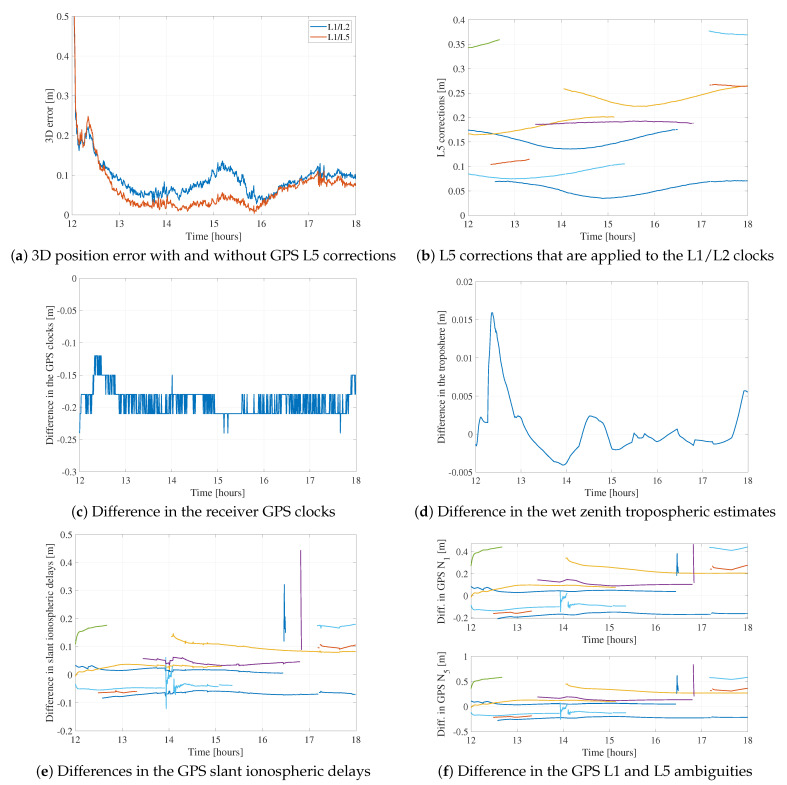
Difference in the estimated GPS-related states when using the GPS L1/L2 and L1/L5 clocks for station BRST on DoY 187 (**a**,**c**–**f**), and applied L5 corrections (**b**).

Figure 11a gives another example of the effect of L5 biases for station BRST on DoY 187 between UTC Hours 12 and 18. The same observations hold as for Figure 10, as 15% and 20% decreases in the horizontal and vertical rms, respectively, are noticed when applying L5 corrections on GEC processing. Therefore, it is clear that the L5 biases have an effect on the position estimates. To better understand the effect of L5 biases on the PPP estimation as a whole, we focus on the BRST dataset. Comparing the GEC results with and without applying the L5 corrections, we notice that the residuals are not affected as they are the same for both solutions, with only millimeter-level differences being seen. Therefore, when not accounting for L5 biases, they do not get absorbed by the residuals. Figure 11 shows the 3D error for station BRST for both solutions, the L5 corrections, and the difference in all the GPS-related states between both solutions. The assumption in these plots is that the only difference between the two solutions is whether the L5 corrections are applied or not. Thus, taking the difference in the estimated states would show the effect of the L5 biases on them.

For instance, the Galileo- and BeiDou-related states are noticed to be very close, within a few millimeters. However, this is not the case for the GPS-related states, as shown in Figure 11. First, the position estimates are affected, as applying the L5 corrections reduces the 3D rms from 8.1 to 5.4 cm. The GPS receiver clock is affected as well, as the difference is 0.2 m on average. That is not the case for the wet zenith tropospheric delay, for which there is less than 2 cm difference between both solutions. The tropospheric estimate is common to all constellations and is estimated as a random walk process, therefore is it more robust to biases than, e.g., the GPS clock or the GPS ambiguities. On the other hand, the slant ionospheric delays and the ambiguities are clearly affected, as decimeter-level satellite-dependent differences are noticeable. It is interesting to note that they follow the same trends as the L5 corrections in Figure 11b. Each color in the correction, ionosphere, and ambiguity sub-figures represents a satellite, each satellite’s color being the same in all sub-figures.

#### 3.3.2. GEC Processing with GPS L5 Corrections Applied

**Figure 12:**
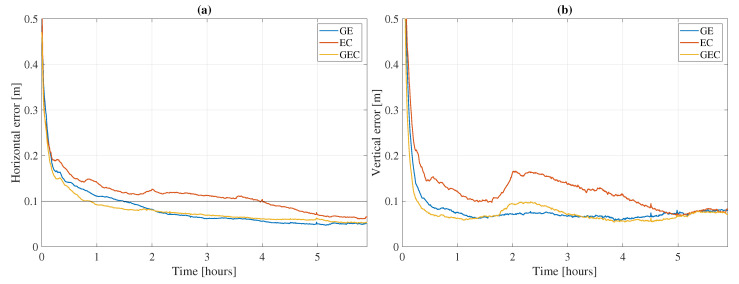
Average horizontal (**a**) and vertical (**b**) errors based on 80 datasets in kinematic mode using [L1,E1,B1]/[L5,E5a,B2a] signals with GE, EC, and GEC. The horizontal black line represents the convergence threshold, defined based on the horizontal error.

The performance of GPS, Galileo, and BeiDou-3 on the same two frequencies 1575.42 and 1176.45 MHz is assessed in this subsection. Figure 12 shows the 100th percentile average horizontal and vertical errors based on processing all 80 chosen datasets. The datasets are processed in three different constellation combinations: GPS + Galileo (GE), Galileo + BeiDou-3 (EC), and GPS + Galileo + BeiDou-3 (GEC). The datasets are processed in kinematic mode. The GPS L5 corrections are applied. The corresponding statistics are summed up in Table 5.

Comparing the GE and EC solutions, it appears that GPS improves the PPP solution more than BeiDou-3 does, since both combinations have Galileo in common and the only difference is the second constellation. On the one hand, it could be due to the number of satellites: even though there are fewer satellites transmitting L5 signals than L2 signals, that number of satellites is still higher than the number of BeiDou-3 satellites. For instance, the average number of processed GPS satellites based on all datasets at all epochs is 4.6 satellites, compared to 3.6 BeiDou-3 satellites (and 7.3 satellites for Galileo). On the other hand, it could be due to mismodeling of, e.g., satellite antenna corrections which are not available for GPS L5 and BeiDou-3 B1/B2a signals or the lower quality of products, as attests the weighting factor in Table 2. Nonetheless, combining all three constellations leads to even better results than with only two-constellation combinations. Indeed, the GEC solution converges faster and reaches lower horizontal and vertical rms than with GE or EC, although the differences between GE and GEC are on the order of centimeters. Note that, when processed in static mode, the three constellation combinations are even closer in performance with very similar convergence times and accuracies.

Although the comparison between the widely used signals shown in Section 3.2 and the results in this subsection is not fair due to the number of satellites not being comparable (e.g., fewer GPS satellites that broadcast L5 signals and no BeiDou-2 with signals on B1 and B2a), the duration of the processing being different (24 h versus 6 h), or the number of processed stations being different, it is interesting to note that the average GEC horizontal and vertical rms in Section 3.2 and in the current section are comparable. Indeed, with the [L1, E1, B1-2]/[L2, E5a, B3] signals for GPS, Galileo, and BeiDou-2/3, the average horizontal and vertical rms are 5.4 and 7.5 cm, respectively, compared to 6.9 and 7.1 cm with the [L1, E1, B1]/[L5, E5a, B2a] signals for GPS, Galileo, and BeiDou-3. This comparison shows that similar performance can be achieved with signals on the same two frequencies for all three constellations, even when fewer satellites are processed.

## 4. Discussion and Future Work

Given the rapid evolution of global constellations, several signals are broadcast by each constellation, some of which are new. In this paper, signals from GPS [L1, L2, L5], Galileo [E1, E5a], BeiDou-2 [B1-2, B3], and BeiDou-3 [B1-2, B1, B2b, B3] are analyzed and their influence on PPP performance is assessed. Satellite products are estimated using DLR’s RETICLE engine, while the user performance is studied through York University’s engine.

A first analysis of the [L1, E1, B1-2]/[L2, E5a, B3] signals, being some of the most logged signals by IGS stations, shows that a combined GPS + Galileo + BeiDou-2/3 solution can reach 10 cm horizontal convergence within 10.5 min at the 95th percentile, with horizontal and vertical rms of 2.1 and 2.3 cm, respectively, in static mode, and 4.6 and 5.9 cm in kinematic mode. The analysis also highlights the effect of the biases existing between both BeiDou generations and the necessity of estimating different receiver clocks for each generation or applying additional corrections.

Thanks to the new BeiDou-3 B1 and B2b signals, all three constellations are broadcasting signals at the same two frequencies: 1575.42 and 1176.45 MHz. The effect of the GPS L5 biases on PPP is assessed first by applying corrections to the L1/L2 clocks. These effects are shown to not only affect the position estimates, but the receiver clock, ambiguities and ionosphere estimates. Accounting for the biases and correcting them leads to clear improvements in the PPP performance with a decrease in both the horizontal and vertical errors. Finally, the performance of all three constellations on the same common frequencies is assessed with horizontal and vertical rms reaching 6.9 and 7.1 cm, respectively, when processed over 6 h in kinematic mode.

Future work will involve un-combined processing of L1, L2, and L5 signals simultaneously instead of dual-frequency processing. With the processing of three frequencies, more biases need to be dealt with, such as inter-frequency biases, although minimal improvements are expected when adding a third frequency for a float solution. Future work will also explore the new additional BeiDou-3 signals B2b and B2.

## Figures and Tables

**Table 1:** PPP processing strategy on the user side.

Parameters	Strategy
Receiver coordinates	Static mode: estimated as constants
	Kinematic mode: 120 km/h process noise
Troposphere	Dry component: GMF model and mapping function [31]
	Wet component: estimated as random walk with process noise of
	2 cm/$\sqrt{h}$ and GMF mapping function
Receiver clock	Estimated as white noise process. One receiver clock per constellation
Ionospheric delays	Slant delays estimated as white noise processes
Ambiguities	Estimated as constants over each continuous arc
Satellite antenna	Corrected for using IGS14 ANTEX corrections [32]
Satellites DCBs	Corrected for using Chinese Academy of Sciences (CAS) products [33]

**Table 2:** Weighting factor $\sigma_{C}$ values for pseudorange measurement weighting (in nanoseconds).

	GPS	Galileo	BeiDou-2	BeiDou-3
$\sigma_{C}$	0.24	0.13	0.86	0.43

**Table 3:** Constellation combinations used in the processing.

Single Constellation	GPS (G)
Dual constellation	GPS + Galileo (GE)
	GPS + BeiDou-2/3 (GC)
	Galileo + BeiDou-2/3 (EC)
Triple constellation	GPS + Galileo + BeiDou-2/3 (GEC)

**Table 4:** Convergence (Conv.) time and horizontal and vertical rms statistics corresponding to the results in Figure 10.

	Conv. Time (mins)	Horizontal rms (cm)	Vertical rms (cm)
L5 corr. not applied	235.5	18.9	17.3
L5 corr. applied	56.5	11.6	10.2

**Table 5:** Convergence (Conv.) time and horizontal and vertical rms statistics corresponding to the results in Figure 12.

	Conv. Time (min)	Horizontal rms (cm)	Vertical rms (cm)
GE	89.0	6.9	7.0
EC	240.5	10.2	11.5
GEC	53.0	6.9	7.1

## Data Availability

The observation data were provided by the Multi-GNSS EXperiment (MGEX) [36] from the International GNSS Service (IGS) and are publicly accessible [37]. L1/L2 satellite orbits and clocks were provided by Deutsches Zentrum für Luft- und Raumfahrt (DLR) and are publicly available as streams. GPS Block-IIF L5 corrections were provided by DLR and are not publicly available. DCB products were provided by the Chinese Academy of Sciences and are publicly available.
